# Research in the time of COVID

**DOI:** 10.1080/13814788.2020.1838184

**Published:** 2020-10-27

**Authors:** Christian Mallen

**Affiliations:** a School of Medicine, Keele University; b NIHR Research Professor in General Practice


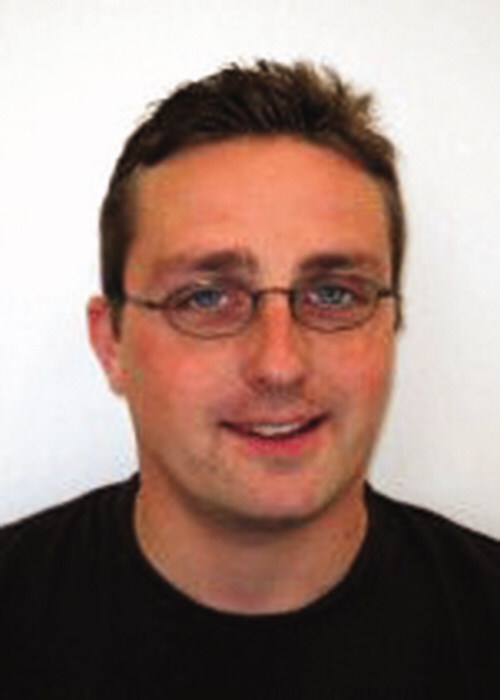
 The impact of the SARS-CoV-2 (COVID-19) is indescribable. On 11 October 2020, there were 37, 259, 238 confirmed cases and 1, 073, 751 deaths recorded across the globe [1]. As such, the pandemic continues to rage and shows little sign of abating. Indeed, with the reality of a second wave upon us, the world seems a very different place today compared with just nine months ago.

The European Journal of General Practice has provided commentary on the impact on clinical services in primary care [2] and rapidly published ‘lessons learned’ from across Europe [3–6]. We have discussed what happens after the first wave and emphasised the widespread impact on our vulnerable patients [7]. In this editorial, we address another impact of the coronavirus pandemic – research.

Primary care has worked hard to establish a strong, vibrant and thriving research environment that addresses the types of clinical problems encountered by people on a daily basis. Whilst our subject matter may not always be the top priority for funders or policymakers, common complaints, such as back pain, constipation and eczema cause real problems for people and impact significantly on their quality of life. Providing a robust evidence base to optimise care in these areas and others is critical for general practice.

COVID has had a huge impact on non-COVID research. Through absolute necessity, huge portfolios of primary care research shut down overnight as health services and research teams were diverted to provide front line clinical care. It was not safe to recruit participants to studies using conventional measures and patients were advised to stay at home whenever possible. As COVID-19 related research, such as vital vaccine studies, have been rapidly funded, delivery teams have reprioritised activity, delaying the restart of research in other clinical areas.

I am not at all critical of these decisions. This had to happen. But what can we learn and how can move forward?

General practice and general practice research are hugely innovative, although sometimes we all need that little nudge to change the way how we do things, especially when the current system seems to work well. Social distancing makes face to face data collection challenging and with no end in sight we need to think differently. We need to invest in technology to allow us to maintain a strong research base for those with long term conditions. As the ageing population (and the research community) become more digitally literate, online data collection becomes increasingly acceptable and has less potential to exclude marginalised groups (although this clearly remains an issue for some). Innovative data collection methods, such as netnography (an extension of ethnographic methods that has been adapted allowing it to be used online), can bring a different perspective to our research, reaching people who have never previously engaged in research. Virtual focus groups often allow more people to contribute, can provide clearer transcripts than audiotaped research and may be more accessible for hard to reach populations. Telephone interviews can be more convenient for participants (and academics) whilst allowing sampling of a more diverse and geographically dispersed population and interventions in clinical trials are being redesigned to delivered remotely. Clearly there are downsides to all these methods but if primary care research is to continue we need to rapidly adapt – and we need our governance structures and funders to be responsive to these essential changes.

General practice also has a significant role to play in supporting ongoing COVID-19 research. Where better to recruit patients to vaccine studies than primary care? We have outstanding relationships with patients who are truly representative of the broader population. Primary care is a convenient setting to recruit participants where practitioners are widely trusted and respected. We have expertise in monitoring adverse events and increasingly sophisticated electronic health records that can rapidly provide accurate and contemporary data on medication, morbidity and key lifestyle demographics to inform research. All of these resources will be invaluable to the ongoing battle against COVID-19.

General practice is also critical to leading research into the long term impacts of coronavirus (‘long COVID’). Increasingly, people who have been diagnosed with COVID-19 are reporting a range of diverse and persistent symptoms including breathlessness, muscle pain, rashes, memory problems and tiredness. Recovery is prolonged for many, with long term consequences unlikely to be fully known for many years. The long term mental health impact of the pandemic is going to be huge. Not only will general practice be at the front line of managing these problems, we will also need to be leading the research in this area in a way only the generalist can.

General practice has adapted rapidly to provide critical health care provision to our patients during the pandemic. Now let’s also think about how we can work differently to support the research which underpins our patient care.
